# Immunotherapy against metastatic bladder cancer by combined administration of granulocyte macrophage‐colony stimulating factor and interleukin‐2 surface modified MB49 bladder cancer stem cells vaccine

**DOI:** 10.1002/cam4.1023

**Published:** 2017-02-16

**Authors:** Chun‐yan Wang, Rui Hua, Li Liu, Xiaomin Zhan, Simei Chen, Song Quan, Qing‐jun Chu, Yong‐tong Zhu

**Affiliations:** ^1^ Department of NeurologyTCM‐Integrated Hospital, Southern Medical UniversityGuangzhouChina; ^2^ Department of Obstetrics and GynecologyCenter for Reproductive MedicineNanfang HospitalSouthern Medical UniversityGuangzhouChina

**Keywords:** Bladder cancer stem cells, granulocyte macrophage‐colony stimulating factor, interleukin‐2, vaccine

## Abstract

In previous studies, it has been shown that the granulocyte macrophage‐colony stimulating factor (GM‐CSF) or interleukin‐2 (IL‐2) surface modified MB49 bladder cancer stem cells (MCSCs) vaccine could induce a specific antitumor immunity and against bladder cancer in mice model respectively. However, whether combined administration of GM‐CSF and IL‐2 could produce specific immune responses to cancer stem cells (CSCs) was uncertain. MCSCs were established and characterized. GM‐CSF and IL‐2 MCSCs vaccines were prepared and bioactivity was evaluated. The therapeutic, protective, specific, and memorial immune response animal experiments were designed. Tumor‐specific cytotoxic T lymphocytes assay, enzyme linked immunosorbent assay, flow cytometry assay were performed to indentify whether vaccine caused an antitumor immunity. Streptavidin (SA)‐GM‐CSF and SA‐IL‐2 MCSCs vaccines were prepared successfully. Such vaccines inhibited the volume of tumor and prolonged the survival of the mice in animal experiments. The express of IgG or IFN‐c, the portion of dendritic cells, CD8^+^ and CD4^+^ T cells were highest in the combined vaccines group than the SA‐GM‐CSF vaccine group, the SA‐IL‐2 vaccine group, the MCSCs group and the PBS group. The combined of GM‐CSF and IL‐2 vaccines could induce better antitumor immunity than a vaccine alone.

## Introduction

Bladder cancer is a most usual urologic cancer in China and the world 1. Following surgery, radiotherapy and chemotherapy, immunotherapy has become the fourth cancer treatment. With the developments in medical research and technology, the feasibility of bladder cancer vaccines for prevention and treatment is increasing 2. In previous studies, it has been shown that the granulocyte macrophage‐colony stimulating factor (GM‐CSF) or interleukin‐2 (IL‐2) surface‐modified MB49 bladder cancer stem cells (MCSCs) vaccine could induce a specific antitumor immunity and against bladder cancer in mice model respectively 3, 4. Cell‐mediated immunity represents the primary approaches by which tumors are attacked by the immune system. Thus far, many reasearchists have developed and optimized the approaches to use antigen‐presenting cells for cancer‐derived antigens in vaccination and immunotherapy for different cancers 5. The interplay of effector cells and cytokines is complex. Cytokines represent the order that permits the immune cells to structure a successful attack. GM‐CSF is produced by NK cells, T lymphocytes, and macrophages and enhances tumor antigen presentation to lymphocytes. IL‐2 enhances the proliferation of both B and T lymphocytes 6.

Recently, Wen et al. constructed an IL2‐GMCSF fusion cytokine, which was able to regulate immune responses against tumors efficiently 7. However, whether combined administration of GM‐CSF and IL‐2 could produce specific immune responses to cancer stem cells (CSCs) was uncertain. Recent findings supported the viewpoint that cancer recurrence and metastasis may be attributed to the incapacity of traditional therapies to eradicate CSCs 8. Our early studies have shown that GM‐CSF or IL‐2 modified CSCs vaccines were able to inhibit metastatic bladder cancer respectively 3, 4. Therefore, theoretically, combined administration of GM‐CSF and IL‐2 will have more potential producing immune response to eradicate CSCs. In this study, in the light of our protein anchor cytokine technology, combined administration of GM‐CSF and IL‐2 surface‐modified MCSCs vaccines were assessed for their role in CSCs metastatic mouse model.

## Materials and Methods

### Establishment and characterizations of MCSCs

MB49 bladder cancer cell was a mouse cell line. MCSCs were obtained from MB49 cells by limited dilution and serum‐free culture medium methods described in early study 8. The serum‐free culture medium consisted of RPMI1640, basic fibroblast growth factor (20 ng/mL), epidermal growth factor (20 ng/mL), B‐27 serum‐free supplement (20 *μ*L/mL), leukemia inhibitory factor (20 ng/mL), and bovine serum albumin (4 *μ*g/mL). MB49 cells were digested, diluted with serum‐free culture medium in a ten‐fold manner several times, and single cells were cultured. Only MCSCs generated clone spheres.

The characterizations of MCSCs were checked in a serious of ways. The expressions of CSCs markers (CD133, CD44) were demonstrated by flow cytometry analysis, western blotting and quantitative polymerase chain reaction. The susceptibility to chemotherapy and proliferative ability were showed by cell counting kit‐8 reagent assay. The migrational and tumorigenic abilities were verified by transwell assay and nude mice.

### GM‐CSF and IL‐2 vaccines preparation

As described previously 3, 4, MCSCs were firstly fixed using 30% ethanol, and following incubated with EZ‐Link Sulfo‐NHS‐LC‐Biotin (Pierce Biotechnology, Rockford, IL). These biotinylated MCSCs were incubated with SA GM‐CSF or IL‐2 fusion protein manufactured in our laboratory. At last, these purified products were the GM‐CSF or IL‐2 surface‐modified MCSCs vaccines.

### Evaluation of GM‐CSF or IL‐2 on the surface of MCSCs

GM‐CSF Vaccine was labeled with FITC anti‐GM‐CSF monoclonal antibody (BD Biosciences Pharmingen, San Diego, CA). IL‐2 Vaccine was labeled with FITC anti‐IL‐2 monoclonal antibody (BD Biosciences Pharmingen). Pure biotinylated cells were set as negative control. These vaccines were evaluated by a BD FACSAria cell sorter.

These vaccines were lysed, and membrane fractions were harvested. The SA‐GM‐CSF and SA‐IL‐2 bioactivities were evaluated by proliferation in bone marrow cells, and the SA‐green fluorescent protein (GFP) was the control group. The bone marrow cells and the membrane fractions were incubated together in 96‐well plates. Cell viability was recorded by the CCK‐8 assay described previously 8.

The level of GM‐CSF or IL‐2 antibody on the vaccine was measured by Western Blotting as described ahead 8. The primary antibodies were anti‐GM‐CSF (Abcam, Cambridge, MA), anti‐ IL‐2 (Abcam) and anti‐*β*‐tubulin (Abcam), while SA‐GFP was set as negative control.

### Ethics statement

All experiments were performed in accordance with China animal protection law and guideline and were approved by the Ethics Committee of Southern Medical University (Contract 1116904).

### Ethics approval and consent to participate

All animal related experiments were authorized by the Ethics Committee of Southern Medical University (Contract 1116904).

### Establishment of subcutaneous and pulmonary metastasis mouse model

C57BL/6 female mice were injected with 1 × 10^5^ MCSCs into the hind leg to produce a subcutaneous model. Additionally, mice were injected with 2 × 10^4^ MCSCs intravenously in the tail vein to produce a pulmonary metastasis model.

### Therapeutic immunotherapy experiment

The subcutaneous and pulmonary metastasis mouse models were established in advance, and then received the different vaccines or other reactants. Mice were randomly divided into five groups and every group consisted of 10 mice. The combined vaccines group was given the combined administration of GM‐CSF and IL‐2 vaccines. The other groups received the GM‐CSF vaccine, IL‐2 vaccine, MCSCs, or phosphate buffer saline (PBS) respectively. The survival time and the subcutaneous tumors volume were recorded.

### Tumor specific cytotoxic T lymphocytes assay

At day 19, splenocytes were isolated from mouse and stimulated by hIL‐2 (R and D systems, Minneapolis, MN) plus the inactivated MCSCs for 5 days. MCSCs served as target cells, and splenocytes served as effector cells. MCSCs and splenocytes were seeded and incubated together; the supernatant was collected after 4 h. The lactate dehydrogenase activity was analyzed by cytotox 96 non‐radioactive cytotoxicity assay (Promega, Madison, WI). Calculation of cytotoxic T lymphocytes assay (CTL) percentage was described in early study 8.

### Enzyme linked immunosorbent assay

At day 19, blood was collected and congealed from mouse. The supernatant was harvested and the concentrations of IgG, or IFN‐*γ* were measured with an enzyme linked immunosorbent assay (ELISA) kit (Abcam). The optical density (OD) value was examined by a microplate reader.

### Flow cytometry assay

At day 19, splenocytes were isolated from mouse, lysed by flow cytometry assay (FCM) lysing solution (Santa Cruz, Dallas, TX), and labeled with PE anti‐CD11c (Biolegend, San Diego, CA) and FITC anti‐CD80 (Biolegend). The ratio of CD11c^+^CD80^+^ cells was measured using a BD FACSAria cell sorter.

At day 19, blood was collected from mouse and labeled with PE anti‐CD4 (eBioscience, San Diego, CA) and FITC anti‐CD8 (eBioscience). The ratio of CD4^+^ and CD8^+^ cells was measured using a BD FACSAria cell sorter.

### Protective immunotherapy experiment

Mice were randomly divided into five groups and every group consisted of 10 mice. The combined vaccines group was given the combined administration of GM‐CSF and IL‐2 vaccine in advance. The other groups received the GM‐CSF vaccine, IL‐2 vaccine, MCSCs, or PBS respectively. At day 20 afterwards, MCSCs attacked them to build the subcutaneous and pulmonary metastasis models. The survival time and the subcutaneous tumors volume were recorded.

### Memory immunotherapy experiment

At day 60 of therapeutic and protective immunotherapy experiment, the tumor free mice or the survived mice in the combined vaccines group were injected MCSCs intravenously again. The blank mice were used as a negative control, and each group had 10 mice. The survival time was recorded.

### Specific immunotherapy experiment

At day 60 of therapeutic and protective immunotherapy experiment, the tumor‐free mice or the survived mice in the combined vaccines group were injected MCSCs subcutaneously in the right hind leg, and prostatic cancer RM‐1 cells in the left hind leg. The subcutaneous tumors volume was recorded.

### Statistical analysis

SPSS19.0 software (SPSS, Inc., Chicago, IL, USA) was used for all statistical analyses. The numeric data was described as the mean value ± SD. The statistical analysis was performed by Students *t*‐test (in 2 groups) or one‐way ANOVA (more than 2 groups). The Kaplan–Meier method was used to analyze survival rates, and the log‐rank test was used to compare the survival rate difference. Differences were set significantly when *P* < 0.05.

## Results

### GM‐CSF and IL‐2 surface modified MCSCs vaccines preparation and evaluation

**Figure 1:**
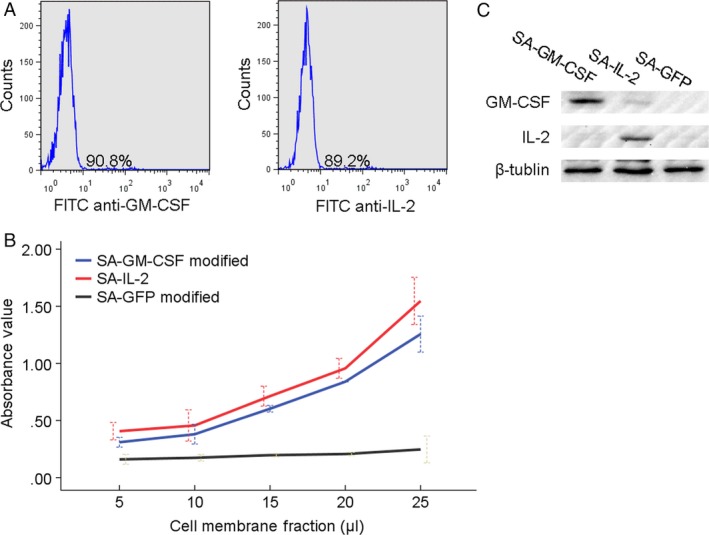
(A) In FCM analysis, the typical image of MCSCs anchored with GM‐CSF and IL‐2. (B) In CCK‐8 assay, the proliferation of bone marrow cells was stimulated by GM‐CSF or IL‐2 in a dosage‐dependent manner. GFP was used as a control group. (C) In WB analysis, GM‐CSF or IL‐2 antibody was abundantly expressed on the vaccine. FCM, flow cytometry assay; MCSCs, MB49 bladder cancer stem cells; GM‐CSF, granulocyte macrophage‐colony stimulating factor; IL‐2, interleukin‐2.

FCM analysis showed that the portion of MCSCs anchored with SA‐GM‐CSF or SA‐IL‐2 was 90.2 ± 1.6% and 89.8 ± 1.5% (Fig. 1A). CCK‐8 analysis showed that the membrane‐bound GM‐CSF or IL‐2 stimulated bone marrow cells proliferation in a dosage‐dependent manner (Fig. 1B). Western Blotting analysis showed that the GM‐CSF or IL‐2 antibody was expressed abundantly (Fig. 1C). These results showed that SA‐GM‐CSF or SA‐IL‐2 could be anchored on MCSCs and retained efficiently biological activity.

### Therapeutic immunotherapy experiment

**Figure 2:**
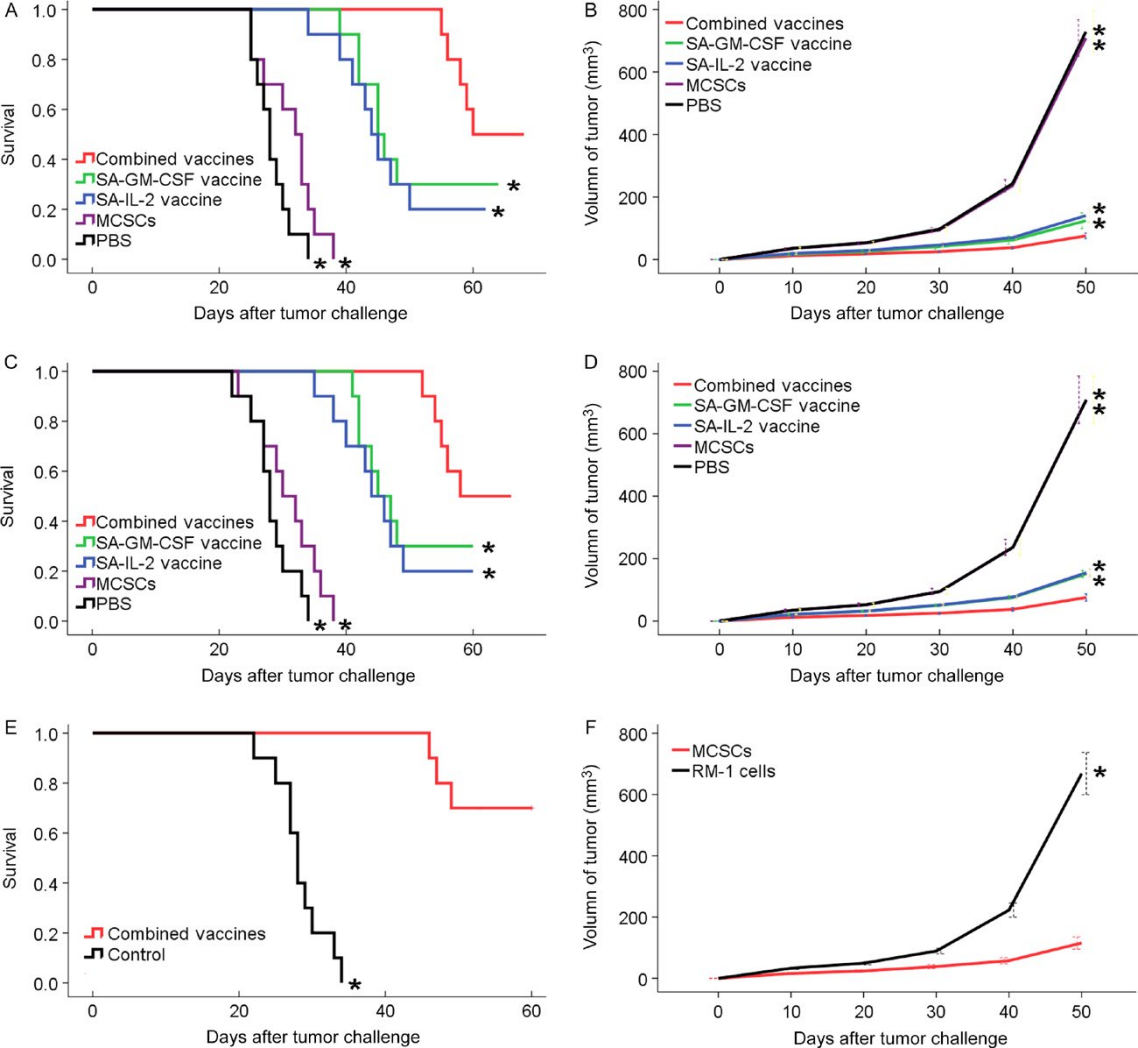
(A) In therapeutic experiment, mice in the combined vaccines group had a longest survival. (B) In therapeutic experiment, mice in the combined vaccines group had a smallest tumor volume trend. (C) In protective experiment, mice in the combined vaccines group had a longest survival. (D) In protective experiment, mice in the combined vaccines group had a smallest tumor volume trend. (E) In memorial experiment, mice in the combined vaccines group had a longer survival. (F) In specific experiment, the legs injected MCSCs had a smaller tumor volume than the legs injected RM‐1 cells. *P < 0.05 (vs. combined vaccines group). MCSCs, MB49 bladder cancer stem cells.

After being treated with the different vaccines or other reactants, significant differences in tumor volume or survival time were observed among the combined vaccines group and the other groups in the subcutaneous or pulmonary metastasis mouse models. The mean tumor volume was 75.6, 124.2, 140.4, 708.8, and 729.0 mm^3^ in the combined vaccines group and the other groups, and the survival rate was 62.8, 49.9, 46.7, 31.2, and 28.3 days respectively. The combined vaccines group exhibited a trend toward significantly smaller tumor volumes or longer survival rate compared with the other control groups (*P* < 0.05; Fig. 2A and B).

### CTL

**Figure 3:**
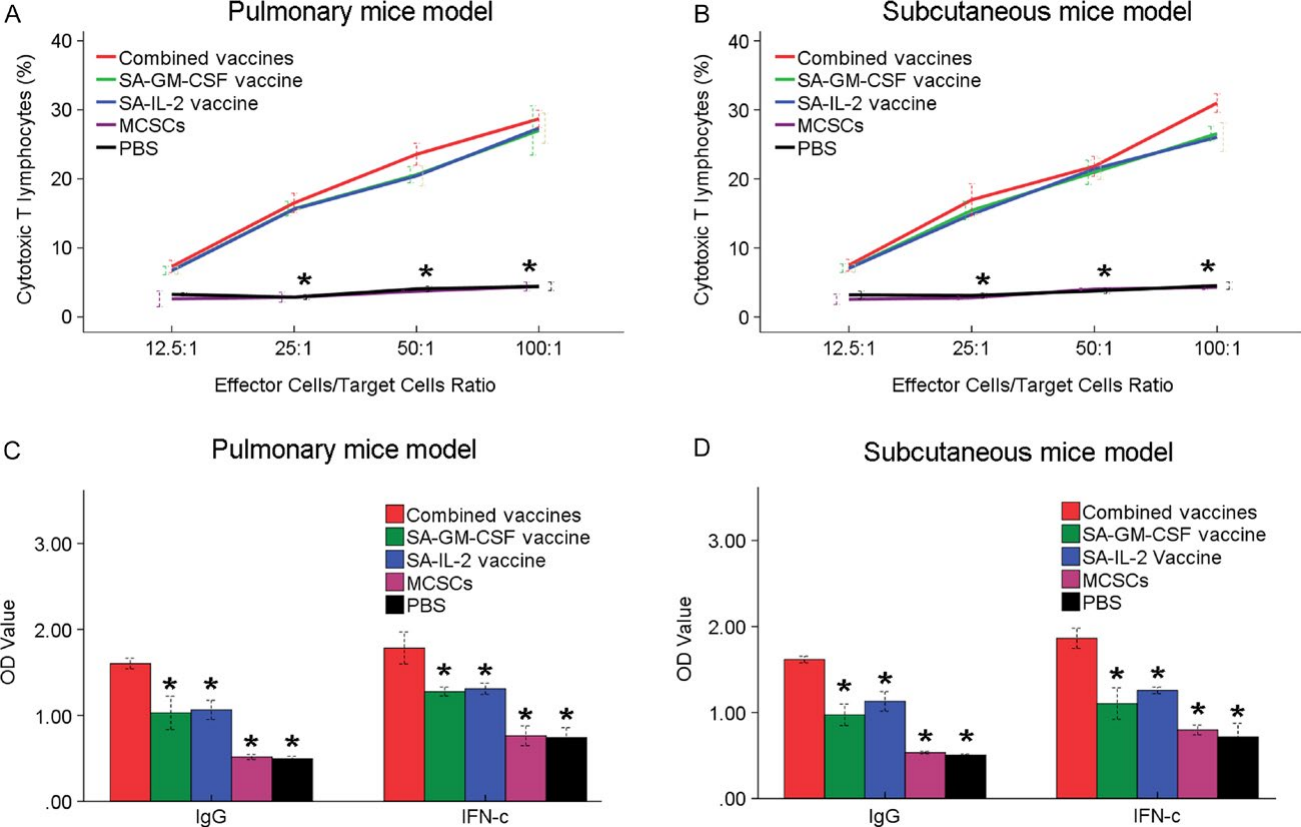
(A) In CTL assay, the portion of CTL in the combined vaccines group was higher than other four groups. (B) In ELISA assay, the serum express of IgG or IFN‐c antibodies in the combined vaccines group was more than other four groups significantly. *P < 0.05 (vs. combined vaccines group). CTL, cytotoxic T lymphocytes assay.

There were significant differences among the combined vaccines group and the other groups at tumor‐specific cytotoxic activities (*P* < 0.05; Fig. 3A). Such results showed that the combined SA‐GM‐CSF and SA‐IL‐2 vaccines could produce a stronger CTL immunity.

### ELISA

The level of IgG or IFN‐c in the combined vaccines group was the highest among five groups (*P* < 0.05; Fig. 3B). Such increased concentrations of IgG or IFN‐c could enhance the antitumor immunity effect in mice.

### FCM

**Figure 4:**
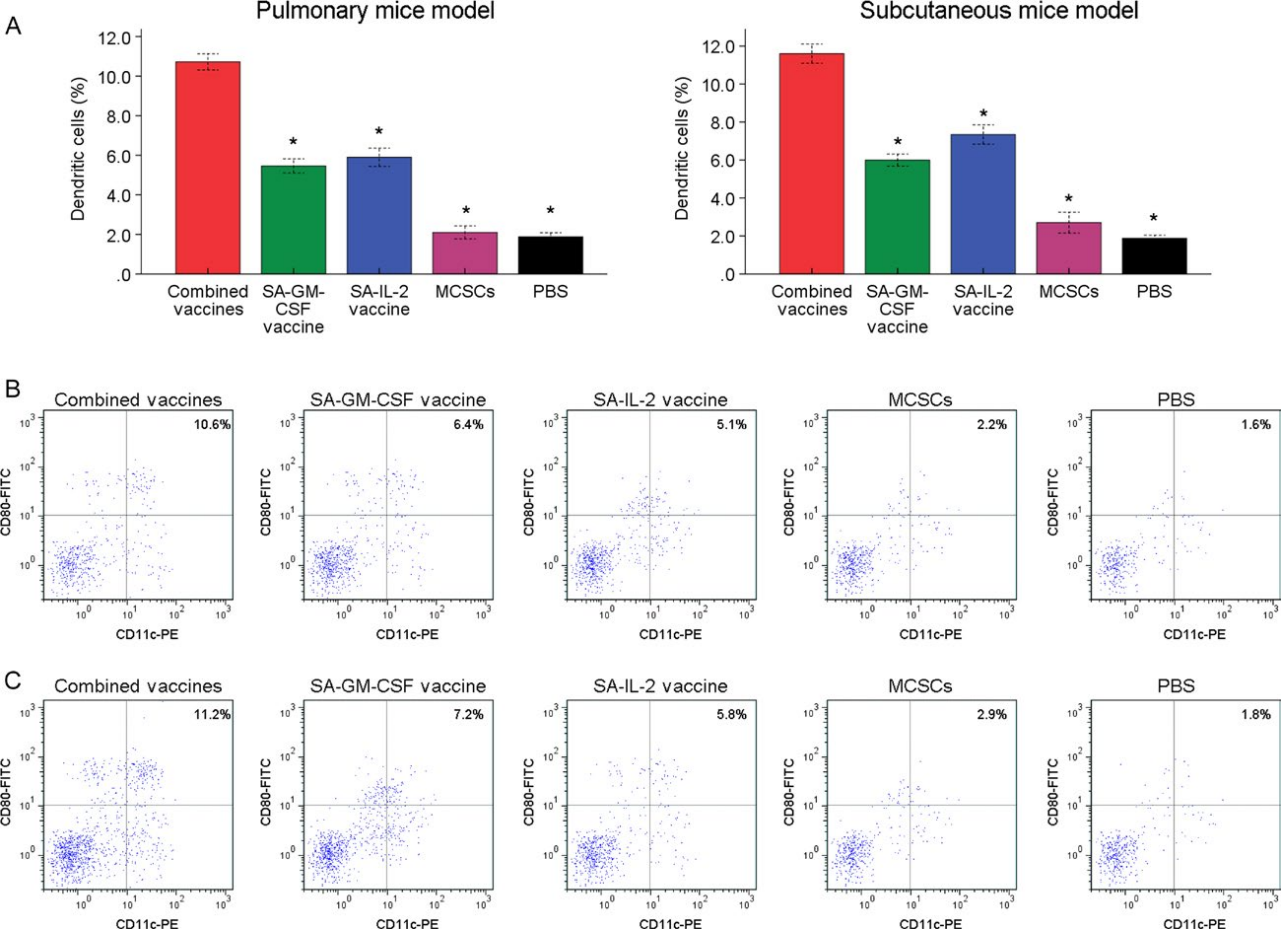
(A) In FCM analysis, the portion of DCs (CD11c^+^ CD80^+^) in the combined vaccines group was more than other four groups significantly. (B) The typical image of DCs in pulmonary mice model. (C) The typical image of DCs in subcutaneous mice model. *P < 0.05 (vs. combined vaccines group). FCM, flow cytometry assay.

**Figure 5:**
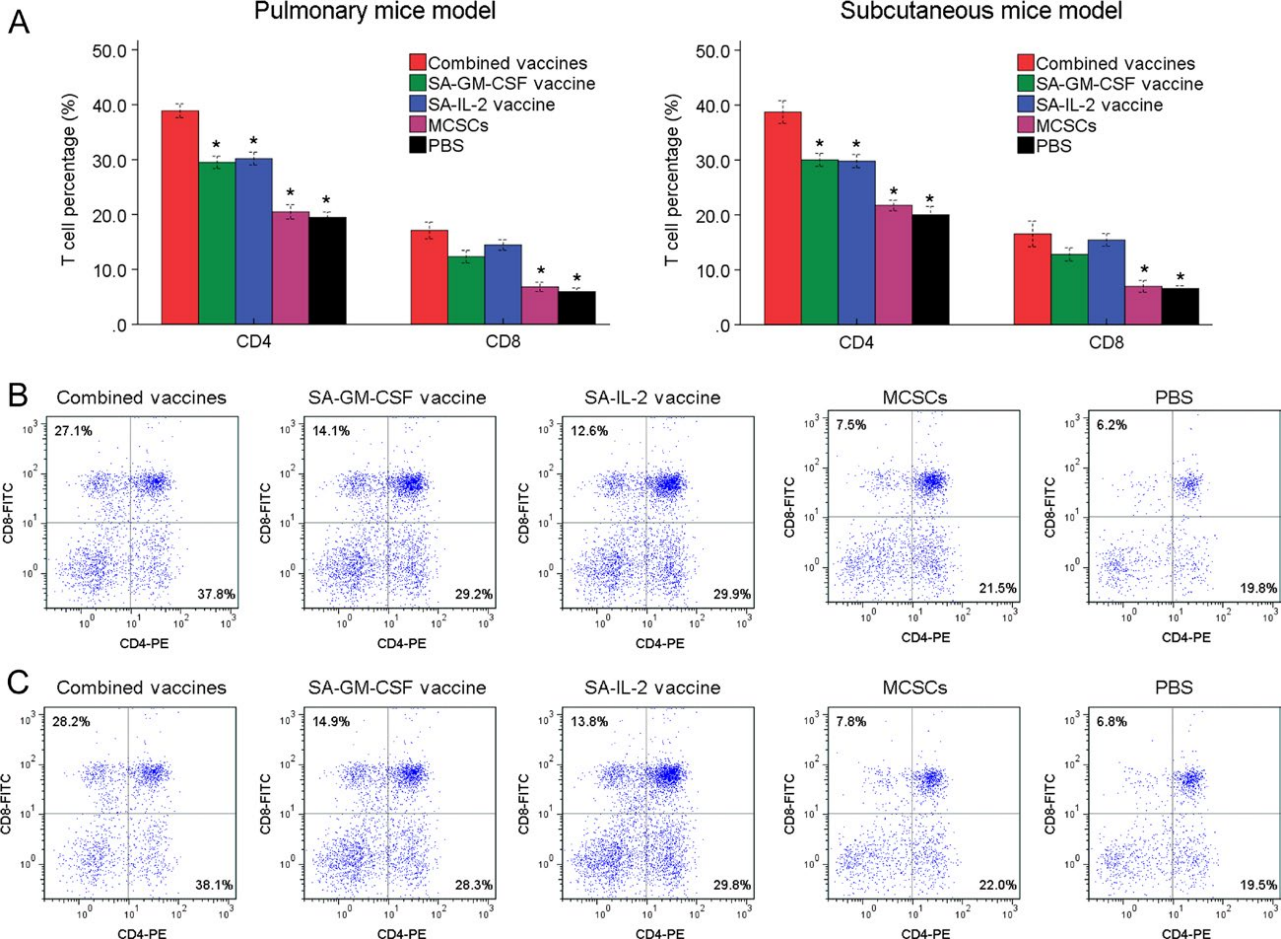
(A) In FCM analysis, the portion of CD8^+^ and CD4^+^ T cells in the combined vaccines group was more than other four groups significantly. (B) The typical image of CD8^+^ and CD4^+^ T cells in pulmonary mice model. (C) The typical image of CD8^+^ and CD4^+^ T cells in subcutaneous mice model. *P < 0.05 (vs. combined vaccines group). FCM, flow cytometry assay.

FCM analysis showed that the number of Dendritic cells (DCs, CD11c^+^CD80^+^) was detected more in the combined vaccines group than other groups (Fig. 4). The proportions of CD4^+^ and CD8^+^ T cells were detected more in the combined vaccines group than other groups (Fig. 5). Such results showed that the combined SA‐GM‐CSF and SA‐IL‐2 vaccines could increase the mature DCs, CD4^+^ and CD8^+^ T cells population.

### Protective immunotherapy experiment

The study on the protective immunotherapy experiment showed that the significant differences in tumor volume or survival time were observed among the combined vaccines group and the other groups. The combined vaccines group had the smallest tumor volumes or longest survival rates (*P* < 0.05; Fig. 2C and D).

### Memory immunotherapy experiment

The combined vaccines group had a longer survival rate than the control group after a second attack of MCSCs (*P* < 0.05; Fig. 2E). Such a result showed that the combined SA‐GM‐CSF and SA‐IL‐2 vaccines could produce a long‐term memory immunity to MCSCs.

### Specific immunotherapy experiment

The right hind legs injected MCSCs had a smaller tumor volume than the left hind legs injected RM‐1 cells (*P* < 0.05; Fig. 2F). Such a result showed that the combined SA‐GM‐CSF and SA‐IL‐2 vaccines could establish a strong tumor specific T cell immunity.

## Discussion

Cancer vaccines have a long historical standing in cancer treatment where they have had a finite success 2. Cancer vaccines concept was based on the assumption that the cancer cells may be the greatest providers of tumor associated antigens and will induce specific anti‐cancer immune responses when used with suitable adjuvant. Except as known tumor antigens, works were underway to recognize new antigens specifically expressed on CSCs 2, 9. Our previous study showed that cultured MCSCs displayed specific CSC properties 8. CSCs vaccines could make full use of inoculators’ own immune system, and may target MCSCs to manage cancer cell relapse, drug resistance, and immune escape 10. In memory immunotherapy experiment, the tumor‐free mice or the survived mice may have complete tumor eradication. So, re‐challenge of these mice would be more convincing of a therapeutic benefit.

The SA‐GM‐CSF and SA‐IL‐2 MCSCs vaccines were produced on the basis of previous vaccine technology, and these vaccines were able to induce antitumor immunity to MCSCs. The use of whole CSCs as vaccines made several advantages 11. First, whole CSCs elicit broad spectrum immune responses to tumor‐associated antigens. Second, the vaccines facilitated cross‐presentation of antigens to CD8^+^ and CD4^+^ T cells. The impact of anticancer immunity partly depends on the function of T lymphocytes. This study showed that the vaccines played a crucial role in the development of DCs, CD8^+^ and CD4^+^ T cells in the immune response, which same with evaluation of effectiveness of DCs based vaccine against CSCs in other model 12.

Antigen‐specific CD8^+^ and CD4^+^ T cells responses were dramatically increasing in the presence of vaccines. This observation was verified in C57BL/6 mice. Although specific innate immune pathways may be activated by MCSCs vaccines, how affects these pathways were unknown. DCs can induce tumor antigen‐specific CTLs, which eradicate tumor cells directly, through the presentation of antigen‐derived peptides on MHC class I 13. IL‐2 was a well‐known multifunctional cytokine that influences different lymphocyte subsets including CTLs, Treg cells and NK cells in immune responses. Vaccines combined with IL‐2 micelle caused intra‐tumoral accumulation of CTLs 13. GM‐CSF was slowly released from GM‐CSF‐encapsulating micelles in the tumor 14. Antigen‐specific CTLs were accumulated and induced efficiently in the tumor.

Some cytokines in vivo would reflex the immune capacity. Similar with other study 15, data from animal experiment confirmed that vaccine with CSCs extended survival of animals and induced higher IFN‐c production. IFN‐c was considered to be the most effective in inhibiting tumor cell growth 16. The level of IgG in vivo increased significantly when using combined vaccines. IgG accounted for more than 80% of total immunoglobulin, and reflected the level of total immunoglobulin. Tumor‐binding IgG combined with DCs could promote potent T cells mediated antitumor immunity 17.

IgG expressed at a low level in normal tissue, but was over‐expressed in many cancers. The level of IgG expression was correlated with low differentiation of cancer cells positively. What's more, IgG promotes cancer cell survival and proliferation in vivo and in vitro 18. IgG was able to serve as a novel target for therapy of cancers and CSCs 19. IFN constituted a cytokine household with pleiotropic effects, and possessed a variety of immunomodulatory activities. IFN‐c was critical for activation of macrophages. The effector T cells were more likely to produce IFN‐c, and central memory T cells were more likely to produce IL‐2. After the beginning of acquired immunity, macrophages were motivated by IFN‐c, mainly from T lymphocytes 20. The mechanism by which IFN‐c mediates CSCs killing was the activation of oxidative burst in macrophages 21.

Some cytokines had an important role in regulating anti‐tumor immune response 22. Cytokines such as GM‐CSF and IL‐2 have been used in cancer treatment protocols for producing a strong anti‐tumor immune response. On the other hand, vaccines using GM‐CSF which secreting cancer cells were showed unpredictable outcomes in some studies 23, 24. Such opposite outcome of GM‐CSF based vaccines for treating cancer may be mainly affected by the overall condition of the immune response. GM‐CSF caused inconsistent outcomes based on the existence of other cytokines 25. Several preclinical studies have revealed that the effect of cancer vaccines was able to enhance by combination with more immunomodulators, such as IL‐2, GM‐CSF, IL‐15, IL‐7, or IFN, and so on 9. This study further demonstrated that combined of GM‐CSF and IL‐2 vaccines could induce better antitumor immunity than a vaccine alone.

There were some limitations that needed to be taken into account. The use of the MCSCs vaccines is not tested against non‐stem cells tumor growth. Our previous studies have shown that MCSCs, compared to non‐stem cells, had higher proliferative abilities, greater migration in vitro, lower susceptibility to chemotherapy, and stronger tumorigenic abilities in vivo. MCSCs vaccines could theoretically induce antitumor function against non‐stem cells. In addition, there have been previous reports of an additive effect of IL‐2 and GM‐CSF in several tumor models. The effects of SA‐GM‐CSF and SA‐IL‐2 MCSCs vaccines maybe related to the cytokines alone. In previous studies, it has been shown that GM‐CSF or IL‐2 did not induce a specific antitumor immunity and against bladder cancer in mice model. However, it is better to include a control group of mice treated with IL‐2 and GM‐CSF without the cells in animal experiments.

However, these vaccines were effective in mice experiments, and not yet performed in clinical trials. These results will need to be confirmed by other researchers, and we will continue to explore the specific mechanism.

## Conclusion

The combined of GM‐CSF and IL‐2 vaccines could induce better antitumor immunity than a vaccine alone.

## Conflict of Interest

The authors declare that there is no conflict of interest that could be perceived as prejudicing the impartiality of the research reported.
